# Robot-assisted laparoscopic hysterectomy for early-stage endometrial cancer with massive uterine leiomyomas: A case report

**DOI:** 10.1016/j.ijscr.2022.107473

**Published:** 2022-08-03

**Authors:** Akiyo Kakibuchi, Fumitake Ito, Tetsuya Kokabu, Hiroyuki Okimura, Osamu Takaoka, Taisuke Mori

**Affiliations:** Department of Obstetrics and Gynecology, Kyoto Prefectural University of Medicine, Graduate School of Medical Science, Kyoto, Japan

**Keywords:** Robot-assisted laparoscopic hysterectomy, Endometrial neoplasms, Leiomyoma, Obesity, Uterus

## Abstract

**Introduction and importance:**

Compared to conventional laparoscopic surgery, robot-assisted surgery enables precise operation, with the aid of high-resolution 3D images and articulated forceps, even in cases where the uterus is very large.

**Case presentation:**

A 48-year-old woman with severe obesity was referred to our hospital with atypical genital bleeding for half a year. She was diagnosed with multiple uterine leiomyomas and early endometrial cancer with presumed advanced stage classification (stage IA). Robot-assisted laparoscopic hysterectomy, bilateral salpingo-oophorectomy, and pelvic lymph node biopsy were performed. Due to the difficulty of removing the uterus transvaginally, the umbilical incision was extended by 7 cm, which allowed the uterine tissue removal without shredding or leakage into the pelvic cavity. The patient was discharged 5 days postoperatively, with no postoperative complications.

**Clinical discussion:**

Robot-assisted surgery has often been used for the management of early-stage endometrial cancer. Robot-assisted laparoscopic hysterectomy has significantly fewer intraoperative and postoperative complications than laparoscopic and abdominal hysterectomy.

**Conclusion:**

Improving this surgical procedure allows for safe and easy robot-assisted uterine malignant tumor removal even in cases where the patient presents with severe obesity and huge uterine leiomyomas.

## Highlights

• Robot-assisted surgery enables precise operation even in cases where the uterus is very large.
• Robot-assisted surgery also enables precise operation even in cases where the patient presents with severe obesity.
• Improving surgical procedure allows for safe and easy robot-assisted uterine malignant tumor removal.

## 1 Introduction and importance

Laparoscopic and robotic surgery is recommended for early-stage endometrial cancer. However, conventional laparoscopy has drawbacks such as limited mobility of laparoscopic instruments, poor ergonomic position for the surgeon, and a steep learning curve [1]. When the uterus is huge as in the case of obesity, laparoscopic surgery may increase the risk. To date, reports of endometrial cancer in a huge uterus that are treated by robot-assisted surgery, are few [2]. Here, we present a case of endometrial cancer in an obese patient with huge uterine leiomyomas that was successfully treated with robot-assisted surgery.

## 2 Case presentation

Informed consent was obtained from the patient, and her identity has been kept confidential. The case has been reported in line with the SCARE 2020 criteria [3].

The patient was a 48-year-old, gravida 0 woman. She visited a nearby obstetrics and gynecology department with complaints of atypical genital bleeding for 6 months prior to presentation. She was diagnosed with endometrioid carcinoma grade 1 by complete endometrial curettage and was referred to our department for further examination and treatment.

**Fig. 1:**
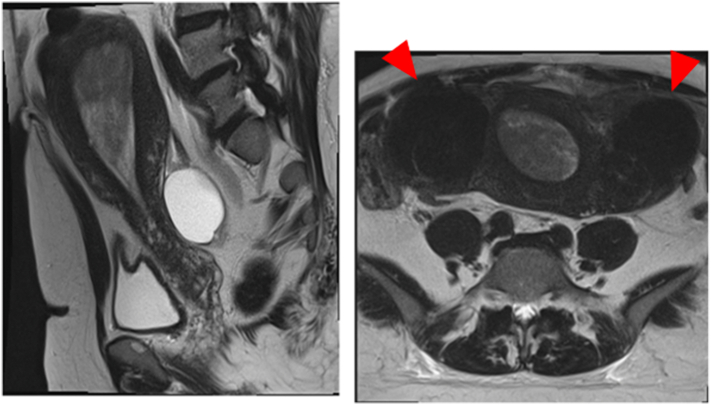
Sagittal T2-weighted MRI: Uterus in a neutral position with a 4-cm endometrial thickening and a 6-cm bifurcated cyst in the left ovary (left). Axial T2-weighted MRI: huge leiomyomas on both sides of the uterine fundus (arrowheads) (right). Abbreviations: MRI, Magnetic resonance imaging.

At the first visit, her body mass index was 40.0 indicating severe obesity. Magnetic resonance imaging showed that the uterus was in a neutral position with a 4-cm endometrial thickening (Fig. 1). Muscular infiltration was not observed. A 6-cm bifurcated cyst was found in the left ovary; the content was serous, and a functional cyst was suspected. Many intramural and interstitial fibroids were found on each side of the uterine fundus, with the largest measuring 7 cm. Positron emission tomography-computed tomography showed fluorodeoxyglucose accumulation of SUVmax 28.4, in a tumor with a major axis of 9 cm occupying the uterine cavity, which was consistent with endometrial cancer. No metastases in the lymph nodes or other organs were observed.

**Fig. 2:**
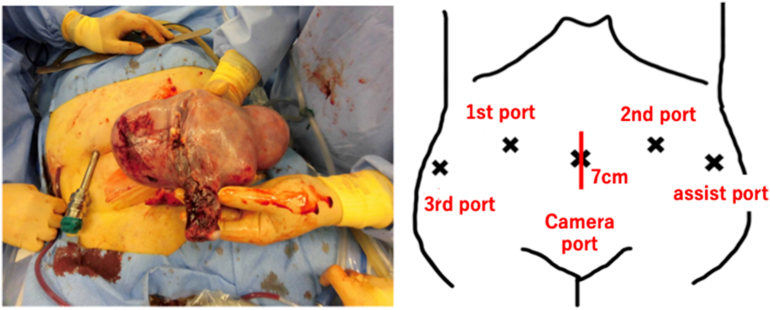
Intraoperative photograph (left). The positions of the five trocars. The incision of the umbilical trocar was extended by 7 cm on both sides, and the uterus was removed without shredding (right).

Thus, a diagnosis of endometrial cancer cT1aN0M0 (FIGO IA) was presumed, and a robot-assisted uterine malignant tumor surgery (hysterectomy, bilateral salpingo-oophorectomy, and bilateral external iliac lymph node biopsy) was performed. Regarding the port arrangement, both the first and second ports were inserted into the higher side of the camera port because of the large uterus. Monopolar scissors, Maryland bipolar forceps, and Cadiere forceps were used for the first, second, and third ports, respectively. A neonatal head-sized, swollen uterus was found in the pelvic cavity. The bilateral fallopian tubes were cauterized, and a manipulator was inserted during laparoscopic observation. After performing a typical hysterectomy and bilateral salpingo-oophorectomy successfully, the lymph nodes in the bilateral external iliac regions were sampled and placed in gloves and retrieved from the assist port. A drain was positioned in the Douglas pouch, and the laparoscopy was completed. After removing the umbilical trocar, the incision was extended by 7 cm to the side. The external uterine orifice was advanced, and the uterus was removed without shredding through it (Fig. 2).

The operative time, including trocar placement as well as robotic docking and closure, was 279 min, the console time was 202 min, the estimated blood loss was 50 g, and the weight of the removed uterus was 1280 g. On histopathological examination, the patient was diagnosed with endometrioid carcinoma G1 because the atypical cells proliferated in a ductal structure, and the solid component was ≤5 %. The tumor size was 96 mm × 60 mm, without muscular or vascular invasion. The histopathological stage was pT1a. The surgical margins were negative. Multiple uterine leiomyomas and adenomyosis were found in the removed uterus. An inclusion cyst was diagnosed in the left ovary. No malignant findings were observed in the external iliac lymph nodes. Since the surgery, no postoperative complications or recurrence has been observed for 3 years.

## 3 Clinical discussion

Robot-assisted surgery has often been used for the management of early-stage endometrial cancer. However, to safely perform the surgery, it is necessary to carefully select the appropriate adaptations for each case. Minimally invasive surgeries for malignant tumors are limited in application due to the method of uterus retrieval, especially when the uterus is large. Thus, robot-assisted surgery for endometrial cancer involving a huge uterus is rare. Here, we have presented a case in which radical surgery was safely performed by devising a surgical procedure for early-stage endometrial cancer complicated by relatively large leiomyomas.

Although laparoscopic surgery is becoming the standard procedure for early-stage endometrial cancer, it may be limited if the uterus is excessively large. To safely introduce robot-assisted surgery in our department, up to 10 cases of uterine myomas and uterine cancer were selected based on examination findings, including magnetic resonance imaging, with a uterine size of ≤10 cm and no suspicion of adhesions. The advantages of robot-assisted surgery over laparoscopic surgery include the lack of restricted movements due to articulation of forceps and the field of view is super-magnified [2], [4]. Moreover, robot-assisted laparoscopic hysterectomy has significantly fewer intraoperative and postoperative complications than laparoscopic and abdominal hysterectomy [5]. In this case, relatively large leiomyomas were present on both sides near the uterine fundus, and for a deeper treatment, uterine artery cauterization was necessary. Robot-assisted surgery allowed for the use of super-magnifying vision and articulated forceps, which is not possible with laparoscopic surgery. This may have contributed to the reduced bleeding.

Many patients with endometrial cancer have severe obesity. In patients with severe obesity undergoing laparoscopic surgeries, the movement of the forceps outside and inside the body is often restricted by the thickened abdominal wall, increasing the load on the surgeon. Thus, a laparoscopic hysterectomy was reported to be more frequently switched to a laparotomy than a robot-assisted surgery [6]. Moreover, the severity of bleeding in robot-assisted surgery is significantly less than that in laparoscopic surgery [6], [7]. Here, despite being obese, the patient only lost a small amount of blood, suggesting the usefulness of robot-assisted surgery.

However, removal of the uterus poses a problem in minimally invasive surgery. For malignant tumors such as endometrial cancer, the uterus is ideally removed as a mass. If the uterus is large or the vagina is narrow, transvaginal removal of the uterus may be difficult, necessitating uterine division for removal. In endometrial cancer, the prognosis is affected by cancer cells spreading into the pelvic cavity when segmenting the uterus [8]. Here, the uterus could not be stored in a collection bag in the pelvic cavity, and the uterus was extruded by extending the umbilical incision to avoid releasing cancer cells into the pelvic cavity. Even larger incision would have been required if the patient was undergone open surgery. As a 7-cm incision was required here, it may be necessary to find a safer and easier method in the future.

Further issues include reducing the time required for difficult cases (*e.g.*, large uterus, adhesions, and patients with obesity). Compared to laparoscopic surgery, robot-assisted surgery often requires performing solo surgery, which may be difficult when communicating with assistants. In robot-assisted surgery, directly manipulating the surgical field is not possible. This effect may be greater in difficult cases and may prolong the operative time. Here, the total operative time of 279 min might be reasonable, given that the uterine weight was 1280 g [9]. As a result, although the surgery took a relatively long time, the surgical wound was significantly shorter than in an open abdominal surgery, and the patient could be discharged from the hospital on the fifth postoperative day, which is the same as in a laparoscopic surgery. In addition, the surgeons are becoming more proficient in robotic surgery through the number of cases, and it will be possible to perform the surgery in a shorter time in the future. It is needed to consider further innovations and cases in which the procedure can be performed with smaller wounds and in less time.

## 4 Conclusion

Herein, robot-assisted surgery for early-stage endometrial cancer in a patient with large uterine leiomyomas and obesity was successfully performed. More reports of robot-assisted surgery in patients with severe obesity will be necessary to further investigate the usefulness of this procedure.

## Declaration of competing interest

The authors have no conflicts of interest relevant to this article.
